# Supplementary material for: Saccharomyces boulardii (CNCM I-745) improves intestinal damage in sepsis by remodeling intestinal flora structure
Source: Front Cell Infect Microbiol. 2025 Jul 31;15:1602792. doi: 10.3389/fcimb.2025.1602792 (PMC12350329; doi:10.3389/fcimb.2025.1602792)
Supplement: Supplementary file 1 [file DataSheet1.zip › summary/summary/7_Differential_analysis/1_diff_abundance/7_Species/Group.DW4_vs_DW0/Group.DW4_vs_DW0_diff_barplot.pdf]

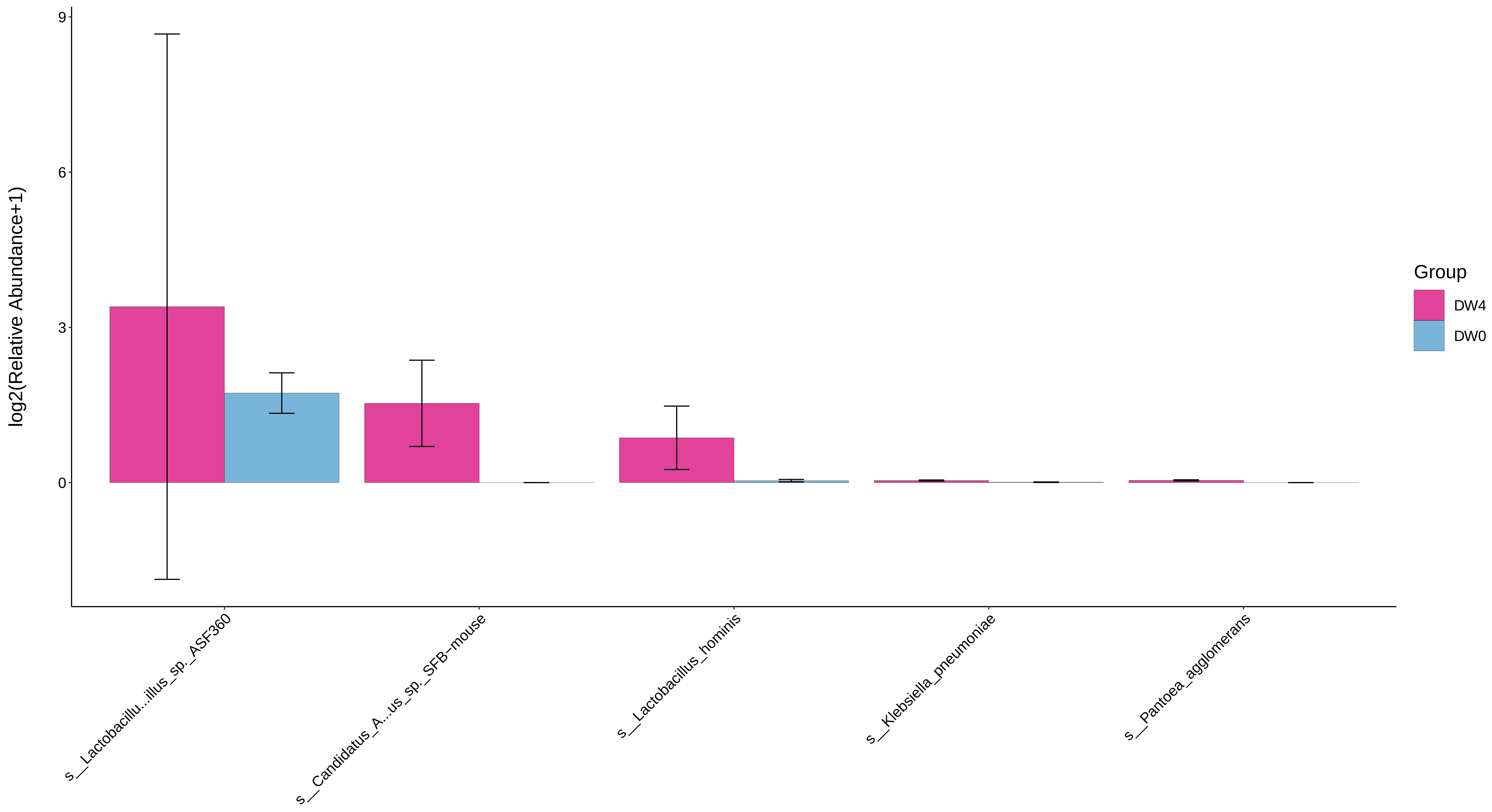

log2(Relative Abundance+1)
9
6
3
0
s__Lactobacillu...illus_sp._ASF360
s__Candidatus_A...us_sp._SFB−mouse
s__Lactobacillus_hominis
s__Klebsiella_pneumoniae
s__Pantoea_agglomerans
Group
DW4
DW0
